# Phase II clinical trial of nirogacestat in patients with relapsed ovarian granulosa cell tumours

**DOI:** 10.1002/ctm2.70568

**Published:** 2026-01-10

**Authors:** Rachel N. Grisham, Elizabeth Hopp, Kathryn Pennington, Robert Holloway, Robert M. Wenham, Pawel Blecharz, Lauren Dockery, Koji Matsuo, Ritu Salani, Mariusz Bidzinski, Patricia Braly, Paul Celano, Thomas Reid, Shelly Seward, Jocelyn Lewis, Mark Johnson, Robert DuBose, Sarah Ahn, Shinta Cheng, Carmelita Alvero, Panagiotis A. Konstantinopoulos

**Affiliations:** ^1^ Department of Medicine Memorial Sloan Kettering Cancer Center New York New York USA; ^2^ Department of Obstetrics and Gynecology Froedtert Hospital and the Medical College of Wisconsin Milwaukee Wisconsin USA; ^3^ Division of Gynecologic Oncology University of Washington Medical Center Seattle Washington USA; ^4^ Gynecologic Oncology Program AdventHealth Medical Group GYN Oncology at Orlando Orlando Florida USA; ^5^ Department of Gynecologic Oncology H. Lee Moffitt Cancer Center & Research Institute Tampa Florida USA; ^6^ Department of Gynecological Oncology Maria Sklodowska–Curie National Research Institute of Oncology Kraków Poland; ^7^ Gynecologic Oncology Section, Obstetrics and Gynecology Department OU Health Stephenson Cancer Center Oklahoma City Oklahoma USA; ^8^ Division of Gynecologic Oncology, Department of Obstetrics and Gynecology University of Southern California Los Angeles California USA; ^9^ Division of Gynecologic Oncology, Department of Obstetrics and Gynecology UCLA Women's Health Clinical Research Center Los Angeles California USA; ^10^ Department of Gynecologic Oncology Maria Sklodowska–Curie National Institute of Oncology Warsaw Poland; ^11^ Women's Cancer Care Covington Louisiana USA; ^12^ Medical Oncology Greater Baltimore Medical Center Towson Maryland USA; ^13^ Gynecologic Oncology Women's Cancer Center at Kettering Kettering Ohio USA; ^14^ Orlando Health, Inc. Orlando Florida USA; ^15^ SpringWorks Therapeutics, Inc. Stamford Connecticut USA; ^16^ AskBio, Research Triangle Park Durham North Carolina USA; ^17^ Department of Medical Oncology Dana Farber Cancer Institute Boston Massachusetts USA

**Keywords:** gamma secretase inhibitors, mutational profiling, NOTCH, tumour recurrence

## Abstract

**Background:**

Adult ovarian granulosa cell tumours (GCT) are the most common subtype of ovarian sex cord‐stromal tumours. Forkhead transcription factor *FOXL2* is required for development and function of normal granulosa cells, including proliferation and ovarian hormone synthesis. A single somatic missense mutation in *FOXL2*, c.402C > G (p.Cys134Trp), has previously been identified in the majority of GCT and is a pathognomonic marker for this tumour type. NOTCH activation contributes to GCT survival in preclinical models, and *NOTCH2* and *NOTCH3* are critical for embryonic development of the ovary and function of the ovarian follicle. Nirogacestat is a potent, selective, noncompetitive inhibitor of gamma secretase, which inhibits NOTCH pathway signalling. Treatment of GCT with nirogacestat was predicted to inhibit granulosa cell survival.

**Methods:**

A Phase II clinical trial was conducted to assess antitumour activity of nirogacestat in adult patients with relapsed/refractory ovarian GCT (NCT05348356). This study enrolled 53 patients; all were evaluable for efficacy and safety. Endpoints included objective response rate by Response Evaluation Criteria in Solid Tumors v1.1 and 6‐month progression‐free survival (PFS6). Fresh or archival tumour samples were analysed for mutational profiling.

**Results:**

Patients received a median of 5 prior lines of therapy (range, 1–13) and a median of 3.7 months of treatment (range, 0–20 months). A decrease in tumour burden was seen in 16 (30%) patients; however, there were no confirmed objective responses. Thirty‐one (58%) patients had stable disease; 18 (34%) had progressive disease. Eleven (21%) patients achieved PFS6. No correlations with disease stability were found with baseline clinical characteristics. All 3 patients who had an activating *NOTCH1* mutation achieved PFS6.

**Conclusions:**

In patients with heavily pretreated GCT, nirogacestat treatment resulted in durable disease stabilisation of at least 7 weeks for 58% of patients, with 21% achieving PFS6, including the 3 patients whose tumours had an activating *NOTCH1* mutation.

**Key points:**

- This Phase II clinical trial of a rare tumour achieved its enrolment target in under 1 year and completed primary analysis within 2 years.
- 87% (46 of 53 patients who received nirogacestat) had fresh or archival biopsies that were analysed by next‐generation sequencing for mutational profiling.
- Of the 3 patients with activating *NOTCH1* mutations, all achieved 6‐month progression‐free survival (PFS6); 8 other patients also achieved PFS6 but did not share a common mutation.

## Graphical abstract

- Evidence suggests NOTCH activation is among the survival and proliferation pathways interacting with *FOXL2* c.402C > G (p.Cys134Trp) mutation in granulosa cell tumours (GCT).
- This Phase II clinical trial of nirogacestat in GCT achieved its enrolment target in < 1 year and primary analysis within 2 years.
- Activating *NOTCH1* mutation was associated with 6‐month progression‐free survival in 3 patients.

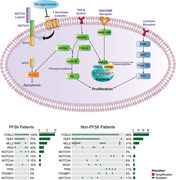

## 1 INTRODUCTION

Adult granulosa cell tumours (GCT) are the most common subtype of ovarian sex cord‐stromal tumours, representing around 5% to 7% of all ovarian cancers.^1^, ^2^ The majority of GCT are detected at an early stage, with symptoms of abnormal uterine bleeding and abdominal and/or pelvic pain commonly reported.^3^, ^4^ The prognosis for patients with GCT is favourable, with surgery often being curative and a 5‐year survival rate for patients with stage 1 disease reported to be approximately 90%.^3^ Risk factors such as advanced stage disease at initial diagnosis and tumour rupture have been associated with poorer prognosis.^3^, ^5^, ^6^ Rates of recurrence are reported between 30% and 50%, and recurrent disease is associated with a decline in survival.^7^, ^8^, ^9^, ^10^ There is variability in time to recurrence, with a median time to relapse of 4 to 6 years, and some occurring more than 10 to 20 years after diagnosis.^3^ There is no standard therapeutic approach for patients with recurrent disease. When feasible, secondary cytoreduction followed by platinum‐based chemotherapy, hormonal agents, and/or radiation is the preferred management strategy.^11^, ^12^, ^13^ Most patients treated with chemotherapy do not show radiographic response, with a cumulative objective response rate (ORR) of 30% reported from studies.^13^ Hormonal therapies, such as aromatase inhibitors, tamoxifen, onapristone XR, or progesterone, have been associated with long‐term responses in a subset of treated patients with recurrent tumours.^14^, ^15^, ^16^, ^17^ Like chemotherapy, however, most patients’ tumours either do not respond or have limited durability of response with a wide variation in progression‐free survival (PFS) noted.^13^, ^15^ One example of this was the PARAGON/ANZGOG 0903 trial, which investigated anastrozole in recurrent/metastatic GCT. In this study, there was a high clinical benefit rate of 79% at the primary endpoint of 12 weeks; however, out of 38 evaluable patients, objective responses were few (1 partial response [PR] at the 12‐week time point and 3 PRs after 12 weeks), and the majority of patients had stable disease with a median PFS of 8.6 months.^18^

Another approach to GCT treatment is antiangiogenic therapy with bevacizumab, which targets vascular endothelial growth factor (VEGF). A study of bevacizumab treatment for GCT in patients who received a median of 2 prior lines of therapy, conducted by the Gynecologic Oncology Group, showed a 17% ORR and a clinical benefit rate of 94% in a small Phase II study.^19^ The ALIENOR/ENGOT‐ov7 trial compared the combination of bevacizumab with paclitaxel to paclitaxel alone in patients with relapsed ovarian sex cord‐stromal tumours, with more than 75% of patients having received ≤2 lines of therapy; the ORR of the combination was 44% compared to 25% ORR with paclitaxel.^20^ The ORR, however, did not correlate with clinical benefit in this patient population as assessed by 6‐month progression‐free survival (PFS6), which was the primary outcome measure. The 72% PFS6 with combination therapy was comparable to the 71% PFS6 with paclitaxel, and the median PFS values (14.9 and 14.7 months, respectively) were virtually the same.^20^ Collectively, the data from these studies highlight that the ORRs achieved with monotherapy and combination therapy in patients with relapsed GCT are variable and do not correlate with improvements in PFS rates. Therefore, there is an unmet medical need in patients with relapsed GCT for therapies that have proven clinical benefits by more than one measure.

Molecular analysis of GCT shows that a single somatic missense mutation in the forkhead transcription factor *FOXL2*, c.402C > G (p.Cys134Trp) is present in 70%–97% of adults with GCT and has value as a diagnostic marker for this tumour type.^21^, ^22^, ^23^, ^24^, ^25^, ^26^ Beyond *FOXL2*, two hotspot mutations in the *TERT* promoter, predominantly the c.124C > T (p.Cys228Thr) mutation, are common and occur in greater than 40% of patients with relapsed disease.^27^, ^28^, ^29^, ^30^ Furthermore, genetic alterations in other genes, including *KMT2D/MLL2, TP53, CDKN2A/B*, and *MTAP*, are also more frequent in patients with relapsed disease, highlighting the emergence of co‐mutations, particularly in tumour suppressor and cell cycle genes, in the relapsed setting.^29^, ^30^, ^31^

NOTCH signalling has been shown to act as a survival pathway in a *FOXL2*‐mutated granulosa tumour cell line (KGN) representative of the adult form of GCTs.^32^ The NOTCH signalling pathway is comprised of four transmembrane receptors (NOTCH1,2,3,4) and five ligands (Jagged [JAG]1,2 and Delta‐like protein [DLL]1,3,4) and plays a critical role in various cellular processes.^33^ Interactions of NOTCH ligands and receptors result in a two‐step enzymatic process, the latter of which results in cleavage of the NOTCH extracellular truncation (NEXT) fragment by gamma secretase, resulting in the release of the NOTCH intracellular domain (NICD) and subsequent translocation into the nucleus to drive downstream gene expression.^34^ Importantly, NOTCH signalling is essential for embryonic development of the ovary and function of the ovarian follicle, including the development of mature oocytes and production of steroid hormones.^35^ Moreover, NOTCH proteins have been shown to be integral for granulosa cell development, proliferation, and function.^35^, ^36^ Ex vivo treatment of ovarian tissue with a gamma secretase inhibitor (GSI) resulted in decreased granulosa cell proliferation and reduced expression of *FOXL2*.^35^, ^37^, ^38^ In addition, over‐expression of NICD2 was able to rescue granulosa cell proliferation in the presence of a GSI.^37^ In separate studies, treatment of the *FOXL2*‐mutated KGN GCT cell line with two different GSIs resulted in decreased proliferation and increased expression of pro‐apoptotic proteins.^32^, ^38^ Overall, these studies highlight a critical role of NOTCH signalling in granulosa cell function and survival and support the investigation of GSIs as a potential therapy for patients with GCTs.

Nirogacestat is an oral, small‐molecule GSI that is approved in the United States for the treatment of adult patients with progressing desmoid tumours who require systemic treatment.^39^, ^40^ Based on the function of *NOTCH* in ovarian follicle development and the decreased survival of *FOXL2*‐mutated cell lines when treated with a GSI, it was predicted that treatment with nirogacestat could inhibit GCT growth. Therefore, a Phase II clinical trial was conducted to assess the antitumour activity of nirogacestat in adult patients with relapsed/refractory ovarian GCT (NCT05348356).

## 2 MATERIALS AND METHODS

### 2.1 Study design

This Phase II trial (NCT05348356) was an international, multicentre, single‐arm, open‐label treatment study to determine the efficacy, safety, tolerability, and pharmacokinetics of nirogacestat in adult patients with relapsed/refractory ovarian GCT. Eligible patients (*N* = 53) were ≥18 years of age, had histologically confirmed recurrent adult‐type GCT of the ovary, had documented radiological evidence of relapse after at least one systemic therapy that was not amenable to surgery or radiation as assessed by the treating physician, and had measurable disease by Response Evaluation Criteria in Solid Tumors (RECIST) v1.1 criteria. Prior systemic therapy was not limited by type (e.g., chemotherapy, hormonal therapy, or anti‐angiogenic therapy), nor was any specific prior line of therapy required. Additionally, there was no limit to the number of prior therapies.

Enrolled patients were administered 150 mg of oral nirogacestat twice daily (BID) continuously in 28‐day cycles. Patients remained on the study treatment until death, disease progression, discontinuation of study treatment for any reason, circumstances that prevented the patient from adhering to the trial protocol, or discontinuation at the request of the patient or investigator. Patients were followed for at least 2 years from the start of study treatment, regardless of treatment discontinuation. Dose reduction to 100 mg BID was included in the protocol for specific grade ≥3 adverse events that persist for ≥3 days, including gastrointestinal, skin, or other clinically significant toxicities.

This trial was conducted in accordance with the ethical principles derived from the Declaration of Helsinki and all applicable laws, regulations, and scientific guidelines. All the patients provided written informed consent before enrolment.

### 2.2 Endpoints and assessments

The primary endpoint was the ORR of nirogacestat treatment in patients with relapsed/refractory GCTs, defined as the proportion of patients with confirmed complete response (CR) and PR using RECIST v1.1 criteria, based on the assessment of the local radiologist. Stable disease was defined as neither meeting criteria for PR nor progressive disease for at least 7 weeks from start of treatment. Magnetic resonance imaging or computed tomographic scans were obtained at screening, at cycles 3, 5, 7, 10, and at every three cycles thereafter. Prespecified secondary endpoints included the estimation of the proportion of patients who had not progressed or died at 6‐month follow‐up (PFS6), estimation of 2‐year overall survival (OS), defined as the proportion of patients who have not died after 2 years of follow‐up after their first dose of nirogacestat, changes from baseline in patient‐reported outcomes (PROs) using the Functional Assessment of Cancer Therapy Ovarian Cancer Symptom Index (FOSI), and determination of duration of response, defined as the time from first assessment of response (CR + PR using RECIST v1.1) to first disease progression defined by RECIST v1.1 or death. Exploratory endpoints included evaluation of next‐generation sequencing status in baseline tumour tissue as well as the change in inhibin A&B, follicle stimulating hormone (FSH), estradiol, CA‐125, and Müllerian inhibiting substance (MIS/AMH) and correlation with response. The first data cut was set based on statistical modelling for mature data of PFS6, as PFS6 is a measure of clinical benefit other than ORR, as shown in the ALIENOR/ENGOT‐ov7 trial.^20^

### 2.3 Mutational profiling

Fresh or archival tumour samples supplied as formalin‐fixed paraffin‐embedded blocks or tumour tissue slides were analysed by next‐generation sequencing (NGS) for mutational profiling using corresponding buffy coat from study visits as reference DNA. Samples were examined using the FoundationOne CDx assay, a US Food and Drug Administration–approved in vitro diagnostic device that targets 324 cancer‐related genes, including *FOXL2*. The CDx assay uses a targeted, high‐throughput, hybridisation‐based capture technology for the detection of copy number alterations, substitutions, and insertions and deletions (indels), as well as readouts on tumour mutational burden and microsatellite instability. Mutational profiling was conducted using the Illumina HiSeq 4000 platform, with >500× median coverage depth and > 99% of exons coverage at >100×.

### 2.4 Data analysis

Primary and secondary efficacy results were generated using SAS software (version 9.4). Statistical analyses of mutational data from the FoundationOne CDx assay and correlations with other clinical parameters were performed in the R statistical computing environment (version 4.4.2). PFS and OS were estimated using the Kaplan‐Meier method. PFS6 is defined as the proportion of patients who had not progressed or died at 6‐month follow‐up, and 2‐year OS rate is defined as the proportion of patients who have not died after 2 years of follow‐up. Associations between patient PFS6 status and gene mutations were assessed using Pearson's χ^2^ test. *p*‐values were computed via Monte Carlo simulation with 10 000 replicates. Values with *p *< .05 were considered significant. Oncoprint‐style mutational heatmaps were produced using ComplexHeatmap::oncoPrint (version 2.22.0).^41^, ^42^

The study hypothesis was that nirogacestat treatment of relapsed/refractory GCT would be considered effective if ORR is at least 30% and ineffective if ORR is ≤15%. An initial accrual goal of 43 participants was selected to ensure sufficient sample size for continuous efficacy monitoring using Bayesian predictive probability. Based on the statistical simulation with sample size of 43 participants, the probability of claiming efficacy is 7% when ORR is 15% and 76% when ORR is 30%.

## 3 RESULTS

### 3.1 Patient characteristics

From September 2022 through June 2023, a total of 53 patients with GCT were enrolled across 16 sites in the United States and Poland. Baseline characteristics and demographic information are outlined in Table 1. At the time of enrolment, the majority of patients were 45 years of age or older (76%) and White (70%). All patients had received at least one prior line of systemic therapy with a median of 5 lines (range, 1 to 13). Twenty‐one (40%) patients received prior treatment with bevacizumab, and 9 (17%) received prior radiation therapy. Eastern Cooperative Oncology Group (ECOG) status at study entry was 0 (74%) or 1 (26%) for all patients. At the time of the data cut, 4 (8%) patients remained on study treatment, and 49 (93%) had discontinued study treatment. Reasons for discontinuation included disease progression (*n* = 35), removal from study due to investigator/sponsor decision (*n* = 8), withdrawal of consent (*n* = 4), death (*n* = 1), and other (*n* = 1). Of the 49 patients who had discontinued treatment, 38 (78%) remained in the ongoing 2‐year survival follow‐up, 1 (2%) died during the treatment period of cardio‐respiratory arrest, which was assessed as not treatment related, 2 (4%) died during the 2‐year follow‐up, and 8 (16%) discontinued from the study.

**TABLE 1:** Patient characteristics.

	Nirogacestat (*N* = 53)
**Age, years**	
Mean (SD)	55.1 (12.9)
Median (min, max)	53.0 (30, 91)
**Age group, years, *n* (%)**	
<45	13 (25)
≥45	40 (76)
**Race, *n* (%)**	
Asian	3 (6)
Black or African American	4 (8)
White	37 (70)
Multiple	2 (4)
Not reported	7 (13)
**Ethnicity, *n* (%)**	
Hispanic or Latino	6 (11)
Non‐Hispanic or Latino	43 (81)
Not reported	1 (2)
Unknown	3 (6)
**Baseline ECOG, *n* (%)** ^†^	
0	39 (74)
1	14 (26)
**Baseline FOSI score**	
Mean (SD)	25.2 (4.7)
Median (min, max)	26.00 (9.0, 32.0)
**Age at time of original OvGCT diagnosis, years**	
Mean (SD)	41.6 (12.3)
Median (min, max)	39.5 (24, 80)
**Relapsed OvGCT disease, *n* (%)**	
Yes	53 (100)
**Time since most recent OvGCT relapsed disease to first dose date, months** ^‡^	
Mean (SD)	2.9 (4.0)
Median (min, max)	1.4 (1, 23)
**Refractory disease, *n* (%)**	
No	37 (70)
Yes	16 (30)
**Time since refractory disease to first dose date, months** ^‡^ **(*n* = 16)**	
Mean (SD)	4.5 (6.2)
Median (min, max)	2.0 (1, 23)
**Number of lines of prior systemic therapy**	
Mean (SD)	5.5 (3.1)
Median (min, max)	5.0 (1, 13)
**Number of lines of prior systemic therapy, *n* (%)**	
1–2	7 (13)
3–4	17 (32)
5–6	12 (23)
7–8	7 (13)
9–10	5 (9)
11–12	4 (8)
>12	1 (2)
**Prior use of bevacizumab, *n* (%)**	
Yes	21 (40)
No	32 (60)

*Note*: Diagnosis dates were imputed where necessary.

Abbreviations: ECOG, Eastern Cooperative Oncology Group; FOSI, Functional Assessment of Cancer Therapy Ovarian Symptom Index; max, maximum; min, minimum; OvGCT, ovarian granulosa cell tumours; SD: standard deviation.

^†^ Baseline was defined as the most recent non‐missing measurement prior to the first administration of study treatment.

^‡^ Time since diagnosis/refractory disease/most recent relapse disease/original relapse disease diagnosis to the first dose date in months. It was calculated as: [dateoffirstdose−dateof(diagnosis/refractorydisease/mostrecentrelapsedisease/originalrelapsediseasediagnosis)+1]/30.4375.

### 3.2 Treatment administration

The median duration of treatment exposure was 3.7 months (range, 0–20 months), and the median (min, max) relative dose intensity was 97% (48, 100). Eighty‐seven per cent of patients received at least 2 cycles of treatment, and 64% received at least 3 cycles.

### 3.3 Toxicity

Treatment‐related adverse events (TRAEs) were reported by 51 (96%) patients, with grade ≥3 events occurring in 36% of patients (Table 2). The most frequently reported grade ≥3 TRAEs (> 1 patient) were diarrhoea (11%), hypokalaemia (8%), rash maculopapular (6%), acute kidney injury (6%), decreased appetite (4%), and dehydration (4%). The most frequently reported TRAEs occurring in ≥20% of patients were diarrhoea (70%), nausea (51%), fatigue (34%), rash maculopapular (32%), vomiting (28%), and hypophosphatemia (28%; Table 3). The most frequent TRAEs leading to dose reduction or interruption (> 10%) included diarrhoea (11%) and rash maculopapular (11%). TRAEs led to discontinuation of treatment in 10 (19%) patients, the most common of which were diarrhoea (8%), fatigue (6%), and nausea (4%).

**TABLE 2:** Summary of grade ≥3 treatment‐related adverse events.

Adverse events classified by system organ class	Patients (%)
Any event	19 (36)
Gastrointestinal disorders	10 (19)
Metabolism and nutrition disorders	6 (11)
Skin and subcutaneous tissue disorders	5 (9)
Investigations	3 (6)
Renal and urinary disorders	3 (6)
Blood and lymphatic system disorders	1 (2)
Cardiac disorders	1 (2)
General disorders and administration site conditions	1 (2)
Immune system disorders	1 (2)
Respiratory, thoracic, and mediastinal disorders	1 (2)

**TABLE 3:** Summary of treatment‐related emergent events that occurred in ≥20% of patients.

Adverse events classified by system organ class	Patients (%)
Gastrointestinal disorders	46 (87)
Skin and subcutaneous tissue disorders	30 (57)
General disorders and administration site conditions	22 (42)
Metabolism and nutrition disorders	21 (40)
Respiratory, thoracic, and mediastinal disorders	16 (30)
Investigations	13 (25)
Nervous system disorders	13 (25)

### 3.4 Activity of nirogacestat

**FIGURE 1:**
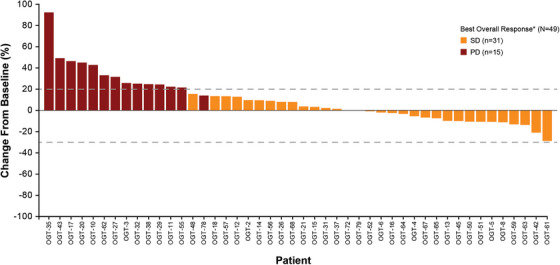
Waterfall plot of per cent change from baseline in tumour size by best overall response. All scans were performed and read locally. Tumour response was defined by RECIST v 1.1. For patients with SD, the minimum per cent change postbaseline was selected. For patients with PD due to at least a 20% increase in the sum of the longest diameters of target lesions, the per cent change from baseline at the time of PD was selected. Per cent change from baseline was not presented for patients with PD due to new lesions (patients OGT‐54 and OGT‐60) or PD in non‐target lesions (patient OGT‐49). *Four patients of the 53 enrolled were missing best overall response data and are not included in this plot. PD, progressive disease; RECIST v1.1, Response Evaluation Criteria in Solid Tumors version 1.1; SD, stable disease.

**FIGURE 2:**
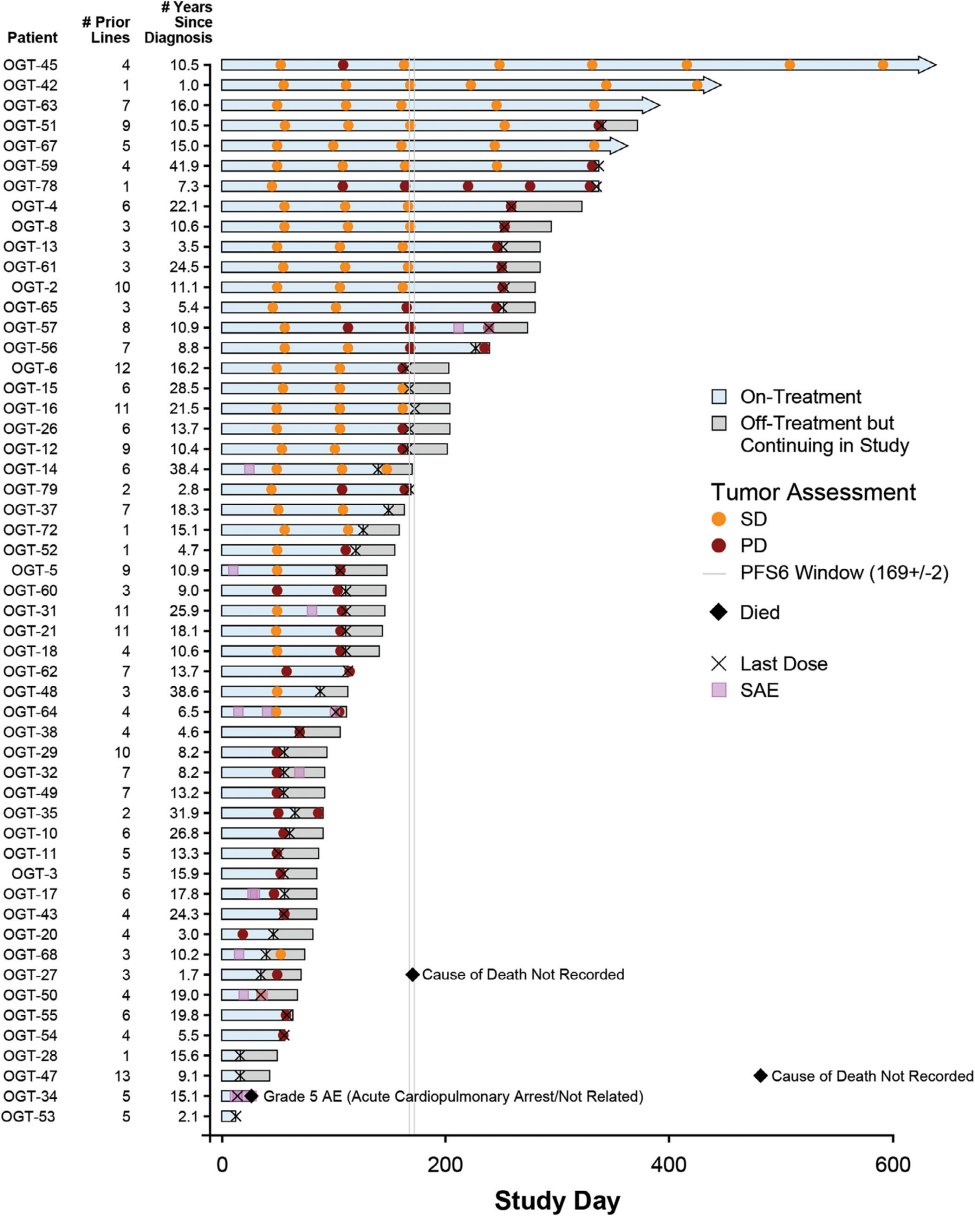
Swimmer plot of patient experience (*N* = 53). AE, adverse event; PD, progressive disease; PFS6, 6‐month progression‐free survival; SAE, serious adverse event; SD, stable disease.

The activity of nirogacestat was analysed in the 53 enrolled patients (Figure 1). At the time of analysis, while a decrease in tumour burden was seen in 16 (30%) patients, no patients achieved a confirmed objective response by RECIST v1.1 criteria, with 31/53 (58%) patients achieving a best response of stable disease (SD). The median PFS was 3.6 months (95% CI, 3.4–5.4), with 11 (21%) patients achieving the pre‐specified secondary endpoint of PFS6 and 3/11 (27%) achieving disease stabilisation for > 9 months (Figure 2). The median OS was not reached with death events in 3 (6%) patients. The proportion of patients who have not died after 2 years of follow‐up was not estimable because not all participants had been on study for 2 years at the time of the data cut‐off.

### 3.5 Patient‐reported outcomes

To ascertain the effect of nirogacestat on ovarian cancer symptoms, the FOSI score was assessed at each clinic visit. The mean and median FOSI scores at baseline were 25.2 and 26.0, respectively (FOSI score ranges from 0 = severely symptomatic to 32 = asymptomatic).

The mean FOSI score was numerically lower relative to baseline through Cycle 5 Day 1 (C5D1), with a median duration of treatment of 3.7 months (data not shown). None of the differences approached the 2 to 3 points that would support clinical significance at any time point. For the patients who achieved PFS6 and provided PRO data beyond C5D1, the mean FOSI score remained numerically lower, suggesting that there was neither improvement nor worsening in ovarian cancer symptoms for those patients who tolerated nirogacestat treatment for a longer duration.

### 3.6 Hormone and tumour markers

FSH receptor and estrogen receptor beta (ERβ) are highly expressed in the majority of GCTs.^43^ The peptide hormones inhibin and anti‐Mullerian hormone (AMH), both produced by the granulosa cells, are potential circulating biomarker candidates that are used for diagnosis and follow‐up of GCT.^44^ Therefore, these measurements, as well as progesterone and luteinising hormone, were also assessed to monitor tumour growth and treatment response. The values of hormone and tumour markers are summarised in Table 4.

**TABLE 4:** Hormone and tumour marker levels.

		PFS6			Non‐PFS6			
Test	Cycle	No.	Median	Q1‐Q3	No.	Median	Q1‐Q3	*p* value
Follicle‐stimulating hormone	Screening	11	7.9	3.2–29.3	40	13.5	4.4–31.9	.69
	C1D1	11	7.1	4.0–28.3	41	6.3	4.0–25.9	.91
	C2D1	10	6.5	2.7–25	38	9.1	4.0–25.7	.95
	C3D1	10	5.8	2–18.9	29	8.7	4.5–20.9	.51
	C4D1	9	6.3	2.1–23.4	21	10.5	3.5–17.3	.93
	C5D1	7	5.3	2.2–23.9	16	7.4	2.9–11.2	1.00
	C6D1	10	6.6	1.63–19.5	12	6.3	3.5–14.3	1.00
Anti‐Mullerian hormone	C1D1	11	79.5	17.0–400.3	41	51.6	21.2–228	.69
	C2D1	10	83.2	26.3–1022.5	38	63.5	26.3–230.3	.63
	C3D1	10	162	18.7–392	29	85.2	35.1–307.8	.85
	C4D1	9	125.2	16.1–1577.5	21	125	62.9–263	.71
	C5D1	7	63.9	36.8–1209.5	16	234.5	76.6–543.5	.62
	C6D1	10	347	55.3–2220.8	12	194	82.5–458.5	.92
Inhibin B	C1D1	11	233	131.5–477.5	41	205	98.8–711	.86
	C2D1	10	296	224–1587	38	198	71.3–733	.52
	C3D1	10	314	89–1148	29	241	129–883	.96
	C4D1	9	416	55–984	21	267.5	192–798.3	.94
	C5D1	7	262	70–913	16	283.5	226.3–1736	.62
	C6D1	10	453	99.5–1392.5	12	477	289.5–1270.8	.54

*Note*: Values below the LLOQ or above the ULOQ of quantification for the assay were substituted with the LLOQ or ULOQ, respectively. Assay units: follicle‐stimulating hormone, mIU/mL; anti‐Mullerian hormone, pmol/L; inhibin B, pg/mL.

Abbreviations: C, cycle; D, day; LLOQ, lower limit of quantification; No, number; PFS6, 6‐month progression‐free survival; Q, quartile; ULOQ, upper limit of quantification.

Analysis of hormone levels showed no FSH spikes for the study population as a whole, and FSH remained close to the first to third quartile range detected at baseline for both PFS6 and non‐PFS6 groups (3.2–29.3 and 4.4 –31.9 miU/mL, respectively) through C6D1. Estrogen and progesterone values were below the limit of detection for almost all patients in both groups throughout treatment. AMH and inhibin B rose markedly through C6D1 for the non‐PFS6 population, consistent with the finding that these two values are markers for tumour growth.^44^

### 3.7 Exploratory biomarker analysis

Fresh or archival biopsies were analysed by NGS for mutational profiling. The purpose of the mutational profiling was twofold: (1) to assess whether the patient population enrolled reflected the mutational profile of patients reported in the literature and (2) to evaluate whether tumour mutational profiles could identify a signature predictive of disease stability. Of the 53 patients who received the study treatment, 46 patients had fresh or archival biopsies that were analysed by NGS for mutational profiling, including 10 (19%) patients who achieved PFS6. The median age of patients with an analysed biopsy was 53 years (range, 30–91). The median time from collection of biopsy to C1D1 of the study was 2.61 years (range, 0.04–13.45). Biopsies were obtained at initial diagnosis or at relapse. Within the 10 PFS6 patient tumour samples that were profiled for molecular alterations, 3 (30%) were from tumours at the time of initial diagnosis, and 7 (70%) were from relapsed tumours. Within the 36 non‐PFS6 patients, 3 (8%) tumour samples were from initial diagnosis, and 33 (92%) were from relapsed tumours.

**FIGURE 3:**
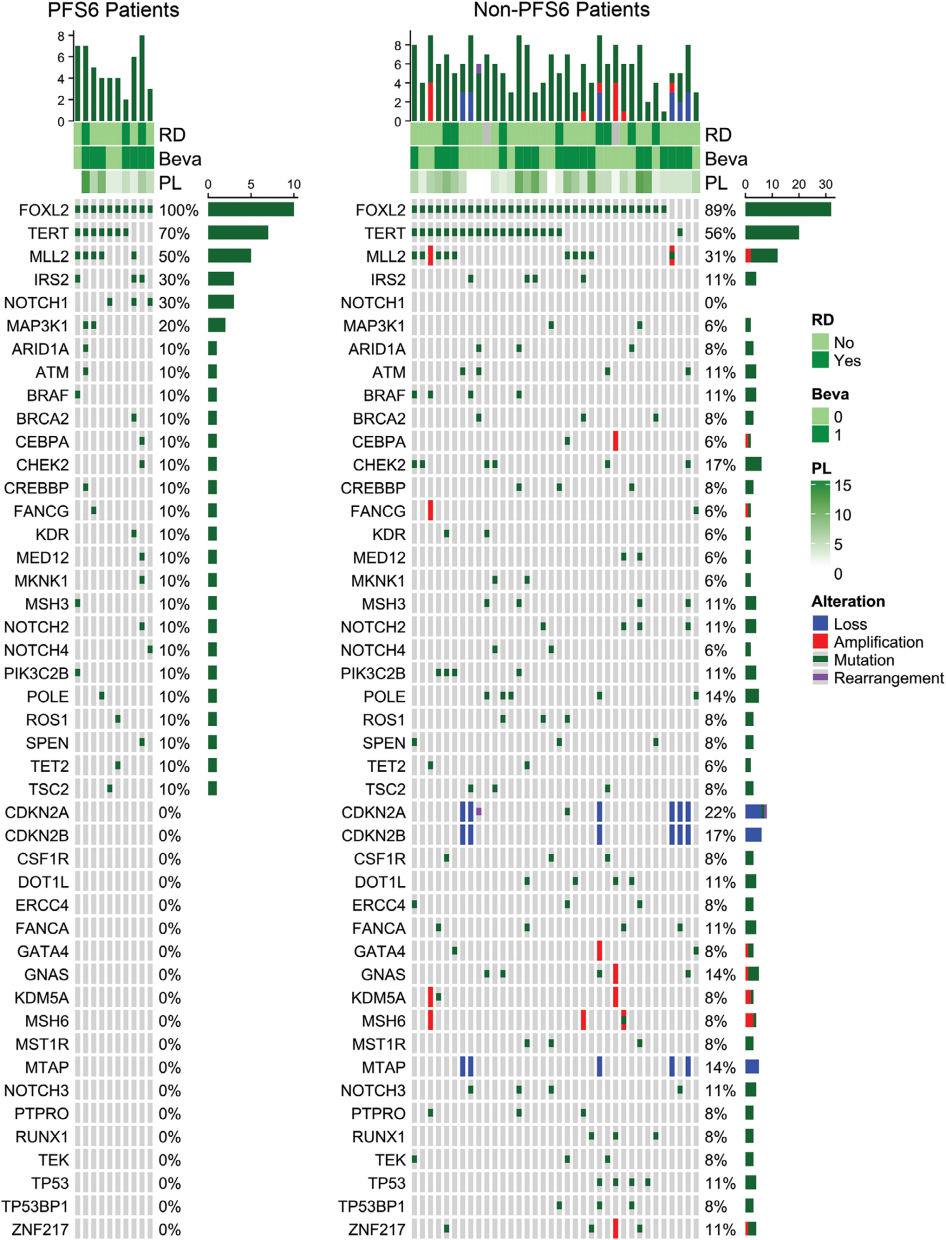
OncoPrint maps depicting alterations observed in > 5% of patients whose baseline biopsies were sequenced and separated by PFS6 status. Genes are ordered by frequency of alteration in the PFS6 group. See Materials and Methods for details on analysis of sequencing data. Beva, bevacizumab; PFS6, 6‐month progression‐free survival; PL, prior lines of therapy; RD, refractory disease.

To determine whether the patient population enrolled was consistent with the mutational profile of GCT patients reported in the literature, we evaluated genes that have been investigated and reported as frequently altered in GCT patients (Table 5 and Figure 3). The data are summarised by alterations in patients who achieved PFS6 and alterations in patients who did not achieve PFS6. *FOXL2* c.402C > G (p.Cys134Trp) was identified in 10/10 (100%) and 32/36 (89%) of PFS6 and non‐PFS6 patients, respectively (*p *= .57). Among the 4 non‐PFS6 patients without a *FOXL2* c.402C > G (p.Cys134Trp) mutation, 3/4 carried deletions in *CDKN2A* and *CDKN2B*, while 2/4 also carried a deletion in *MTAP*. Furthermore, among those 4 non‐PFS6 patients, one also harboured a c.124C > T *TERT* promoter mutation, a second harboured a mutation in *CHEK2*, and a third harboured a missense mutation and amplification in *MLL2* (Figure 3).

**TABLE 5:** Molecular characteristics of OvGCT patients stratified by PFS6 status.

	PFS6 (*n* = 10)					Non‐PFS6 (*n* = 36)					
Gene	No. patients with alteration	Gene alteration percentage	Alteration	Count	Percentage	No. patients with alteration	Gene alteration percentage	Alteration	Count	Percentage	*p* value
*FOXL2*	10	100%	Wild‐type	0	0%	32	89%	Wild‐type	4	11%	.57
			C134W	10	100%			C134W	30	83%	
								C134W, A308V	1		
								C134W, A234del	1	3%	
*TERT*	7	70%	Wild‐type	3	30%	20	56%	Wild‐type	16	44%	.49
			Promoter ‐124C > T	5	50%			Promoter ‐124C > T	16	44%	
			Promoter ‐146C > T	1	10%			Promoter ‐146C > T	4	11%	
			Promoter ‐146C > T, promoter ‐124C > T	1	10%						
*MLL2*	5	50%	Wild‐type	5	50%	11	31%	Wild‐type	25	69%	.28
			E1080Q	1	10%			P2515S	1	3%	
			E1426*	1	10%			I5497del	1	3%	
			R2771*	1	10%			Amplification	1	3%	
			I1200fs*7	1	10%			T4890I	1	3%	
			P692T	1	10%			V3567fs*21	1	3%	
								Amplification, T1246M	1	3%	
								A2432fs*53	1	3%	
								Q356*	1	3%	
								Q3724_Q3725insLQ	1	3%	
								D4530N	1	3%	
								P692T	1	3%	
*CHEK2*	1	10%	Wild‐type	9	90%	6	17%	Wild‐type	30	83%	.68
			T367fs*15	1	10%			I157T	3	8%	
								Splice site 593‐1G > T	1	3%	
								Splice site 683+2T > C	1	3%	
								T367fs*15	1	3%	
*TP53*	0	0%	Wild‐type	10	100%	4	11%	Wild‐type	32	89%	.56
								C275Y	1	3%	
								C141Y	1	3%	
								H193R	1	3%	
								R175H	1	3%	
*NOTCH1*	3	30%	Wild‐type	7	70%	0	0%	Wild‐type	36	100%	.0079
			D869N	1	10%						
			D469E	1	10%						
			R955H	1	10%						

*Note*: Associations between single gene alteration status and PFS6 status were performed utilising Pearson's χ^2^ test.

Abbreviations: No, number; OvGCT, ovarian granulosa cell tumours; PFS6, 6‐month progression‐free survival.

By frequency, the most common mutations outside of *FOXL2* occurred in the *TERT* promoter. There were no significant differences in the frequency of *TERT* mutations identified in the PFS6 (70%) versus non‐PFS6 (56%) groups (*p *= .49), with the c.124C > T mutation being the predominant mutation in both groups. The frequency of alterations in the *MLL2* gene was likewise not statistically different in the PFS6 (50%) versus non‐PFS6 (31%) groups (*p *= .28), and mutations with known or likely oncogenic significance were identified at the same frequency within both groups. Somatic mutations in *CHEK2* were identified in 1/10 (10%) PFS6 and 6/36 (17%) non‐PFS6 patients, respectively (*p *= .68). The presence of a *TP53* mutation was noted only in 4 (11%) non‐PFS6 patients. The *FOXL2*, *TERT*, *MLL2*, *CHEK2*, and *TP53* mutations tested by χ^2^ were selected because of their relevance to GCT biology, and *NOTCH1* (described below) was selected for testing because it is a direct biological target of gamma secretase.^33^ The nominal *p* values associated with alterations in these individual genes as tested by χ^2^ methodology must be interpreted with caution because of the rarity of this disease resulting in the small sample size (potentially resulting in χ^2^ cell counts of < 5 in some cases) and the mix of archival and fresh biopsies.

### 3.8 Evaluation of NOTCH

Among the 46 biopsy samples evaluated, 13 (28%) patients had alterations in *NOTCH* genes. These patients consisted of 4 (31%) PFS6 patients and 9 (69%) non‐PFS6 patients. Eleven (24%) patients possessed a single altered *NOTCH* gene, and 2 (4%) patients possessed alterations in 2 *NOTCH* genes (*NOTCH1* and *NOTCH4* in one patient and *NOTCH3* and *NOTCH4* in the second).

Fifteen unique alterations were identified. Of the 15 *NOTCH* alterations, 1 encoded a nonsense mutation in the intracellular portion of *NOTCH2* that could not be mapped to a specific domain. The remaining 14 alterations encoded for a missense mutation.

*NOTCH1* mutations were found only in the subset of patients who achieved PFS6 (3/10; 30%) and mapped to the extracellular EGF‐like domains. One of these patients had the *NOTCH1/NOTCH4* double alteration. This segregation of alterations in *NOTCH1* to patients achieving PFS6 was statistically significant (*p *= .0079, Table 5); this was the only gene of the *NOTCH* paralogs or other genes examined to reach significance between PFS6 and non‐PFS6 patients.

Five alterations in *NOTCH2* were observed, with 4 of the 5 alterations found in non‐PFS6 patients and one in a PFS6 patient. With respect to the predicted impact of the mutation on the function of the protein, in addition to the nonsense *NOTCH2* mutation in the non‐PFS6 patient, a missense mutation in *NOTCH2* in a PFS6 patient with refractory disease mapped to the ankyrin repeat domain that was predicted by the Protein Analysis Through Evolutionary Relationships‐Position Specific Evolutionary Preservation (PANTHER‐PSEP) algorithm^45^ to have an adverse functional impact (‘damaging’ in the PANTHER terminology) on the protein.

Four *NOTCH3* alterations were observed and found only in patients who did not achieve PFS6 (4/36; 11%). One of these four patients had the *NOTCH3/NOTCH4* alteration. These mutations mapped to both the extracellular and intracellular domains of *NOTCH3*.

Alterations in *NOTCH4* were identified in both PFS6 and non‐PFS6 patients. *NOTCH4* alterations were observed in 2 (6%) of the non‐PFS6 patients and 1 (10%) PFS6 patient. Like *NOTCH1*, the *NOTCH4* alterations mapped to the extracellular EGF‐like domains. Additional details on the predicted effects of the alterations on each of the NOTCH proteins are given in Table 6.

**TABLE 6:** *NOTCH* mutational status of 13 patients, of which 2 patients were identified to harbour mutations in > 1 NOTCH protein.

Refractory	PFS6	Tx > 9 months	Gene	Protein change	Mutation	Location	Domain	PANTHER‐PSEP prediction
N	N	N	*NOTCH2*	R1786*	Nonsense	Intracellular	Unspecified	
N	N	N	*NOTCH2*	T2327A	Missense	Intracellular	Transactivation	Probably benign
N	N	N	*NOTCH2*	T563N	Missense	Extracellular	Unspecified	Probably benign
N	N	N	*NOTCH3*	N1588H	Missense	Intracellular	Ankyrin repeat region	Probably damaging
			*NOTCH4*	R230H	Missense	Extracellular	EGF‐like 5	Probably benign
N	N	N	*NOTCH3*	R1190H	Missense	Extracellular	EGF‐like 30	Probably damaging
N	N	N	*NOTCH2*	V1667I	Missense	Extracellular	Heterodimerisation	Probably benign
N	N	N	*NOTCH4*	Q63H	Missense	Extracellular	EGF‐like 1	Probably benign
N	N	N	*NOTCH3*	P2178S	Missense	Intracellular	PEST	Probably benign
N	N	N	*NOTCH3*	V560M	Missense	Extracellular	EGF‐like 14	Possibly damaging
N	Y	N	*NOTCH1*	D869N	Missense	Extracellular	EGF‐like 23	Probably damaging
N	Y	N	*NOTCH1*	D469E	Missense	Extracellular	EGF‐like 12	Probably damaging
Y	Y	Y	*NOTCH2*	R1953C	Missense	Intracellular	Ankyrin repeat region	Probably damaging
N	Y	Y	*NOTCH1*	R955H	Missense	Extracellular	EGF‐like 25	Possibly damaging
			*NOTCH4*	G228S	Missense	Extracellular	EGF‐like 1	Probably benign

Abbreviations: EGF, epidermal growth factor; PANTHER‐PSEP, Protein Analysis Through Evolutionary Relationships‐Position Specific Evolutionary Preservation; PEST, protein domain rich in proline (P), glutamic acid (E), serine (S), and threonine (T); PFS6, 6‐month progression‐free survival; Tx, treatment.

## 4 DISCUSSION

Based on the underlying function of *NOTCH* in ovarian follicle development and the role of *NOTCH* in granulosa cell survival, this Phase II clinical trial was conducted to assess the antitumour activity of nirogacestat in adult patients with relapsed/refractory ovarian GCT (NCT05348356). The enrolled population was reflective of the variability within this patient population with respect to duration of disease, disease burden, ECOG status, and number of prior treatment regimens. There was, however, a low proportion of Black patients (8%) enrolled in this study, which does not reflect the higher incidence reported in Black patients compared with White patients (0.44 vs. 0.18 per 100 000, respectively).^46^ This possibly presents a bias, as the enrolled patient population was not representative of the documented frequency of ovarian GCT in the general US population. Treatment with nirogacestat resulted in SD for a subset of patients, with 11 of 53 (21%) evaluable patients achieving the secondary endpoint of 6‐month PFS. This PFS6 rate is notably lower than the 71% to 72% rate detected in the bevacizumab plus paclitaxel combination therapy, which enrolled a population of 85% verified GCT patients with > 75% of patients having received 2 lines of therapy or fewer.^20^ However, this PFS6 rate is similar to the PFS6 rate reported in a study of onapristone extended‐release monotherapy in progesterone receptor positive GCT (21.4%) in which patients received a median of 2 (range, 1–6) prior lines of chemotherapy and a median of 1 (range, 0–8) prior lines of hormonal therapy^16^ and had no responses by RECIST. A combination of onapristone extended release with anastrozole in progesterone receptor positive GCT had similar results of 28.6% PFS6.^17^ Of note, the median of 5 lines of prior therapy for the population enrolled in this study was higher than the median number of lines of therapy for the populations in these other trials, making comparison of outcomes challenging.^16^, ^17^, ^20^ PRO results through C5 of this study, as measured by the FOSI, suggest that nirogacestat treatment neither improved nor worsened ovarian cancer symptoms, and the reported TRAEs were consistent with the overall experience of nirogacestat in solid tumours.^39^, ^47^, ^48^

Mutational profiling from 46 evaluable patients was conducted to determine whether correlations could be made with achieving PFS6. Of particular interest was the distribution of *NOTCH* mutations. We observed that alterations in *NOTCH1* were found only in patients who achieved PFS6 (3/10; 30%), whereas alterations in *NOTCH3* were found only in patients who did not achieve PFS6 (4/36; 11%). Further, all but one of the alterations identified in *NOTCH3* are predicted to be pathogenic according to PANTHER‐PSEP, a database that estimates the functional effect of a mutation on a protein based on an evolutionary conservation score.^45^ *NOTCH* alterations were also identified in 2 out of 3 PFS6 patients who remained on study for > 9 months, one of whom carried a missense mutation in the ankyrin repeat region of *NOTCH2* and the other with missense mutations in the extracellular domains of both *NOTCH1 and NOTCH4*. Of note, all 3 of the PFS6 patients with *NOTCH1* alterations had received ≥3 prior lines of therapy, and the remaining PFS6 patients had a wide range of prior treatment lines.

There are contrasting reports on the role of *NOTCH1* variants in breast cancer, irrespective of oncogenic phenotype and their value in predicting sensitivity to GSIs.^49^, ^50^ Our study attempted to evaluate *NOTCH* activity by detecting NICD in biopsy samples by immunohistochemistry. However, NICD could not be detected in wild‐type or mutant granulosa cells during assay validation. Hence, we were unable to determine whether the alterations identified had altered NOTCH signalling that could be attenuated by nirogacestat treatment. Future studies could evaluate canonical *NOTCH* target genes by RNA sequencing to assess NOTCH signalling. Additionally, evaluation of gene alterations in the NOTCH signalling pathway by whole exome sequencing may provide insight as to whether tumours harbour activating alterations that are unaffected by gamma secretase inhibition by nirogacestat.

Dysregulated WNT/β‐catenin signalling has been implicated in the development of GCT.^51^ There is evidence that desmoid tumours resulting from the growth of mesenchymal stromal cells in a wound healing setting are associated with deregulated WNT signalling due to APC loss.^52^ In this study, alterations in β‐catenin (*CTNNB1*) were not identified by NGS. Moreover, frequencies of mutations in the *TERT* promoter, *MLL2*, *CHEK2*, and *TP53*—genes that have been reported to be associated with disease recurrence and poor survival in GCT^27^, ^28^, ^29^, ^31^, ^53^, ^54^—did not differ between patients who achieved PFS6 and those who did not. Interpretation of this data may be confounded by the variability in archival biopsies taken at diagnosis or at the time of relapse.

This study achieved its enrolment target in under 1 year, with the majority of patients having tumour samples evaluable for mutational analysis. Comprehensive genomic analysis of known proliferation and survival genes of GCT was performed. We have shown that there is no single survival gene that is consistently mutated. An interesting observation is that the 3 patients who had an activating mutation in *NOTCH1* achieved PFS6. Eight additional patients who also achieved PFS6 did not have a shared mutation. There remains a high unmet need in this patient population for treatment in the relapse setting, and it is likely that combination therapy targeting more than one biological pathway may be needed for improved outcomes.

## AUTHOR CONTRIBUTIONS

All authors made substantial contributions to the conception or design of the work; drafted the work/revised it critically; approved the version to be published; and agree to be accountable for all aspects of the work.

## CONFLICT OF INTEREST STATEMENT

R.N.G. has received consulting fees from Verastem, AstraZeneca, GlaxoSmithKline, Corcept, Incyte, and Genmab; has received payment or honoraria from GOG Partners; and has held a leadership or fiduciary role for the NCCN Guidelines. E.H. has served on an advisory board for Immunogen. R.H. has received consulting fees from GlaxoSmithKline, Genelux, and AbbVie; has received payment or honoraria for GlaxoSmithKline, Merck, Natera, and AstraZeneca; and owns stock options with Genelux. R.W. has received grants or contracts from OnTarget Lab and Anixa Biosciences; has received consulting fees from Merck, Genentech, Mural Oncology, GlaxoSmithKline/Tesaro, AstraZeneca, AbbVie, Legend Biotech, Regeneron, Seagen, Shattuck Labs, Eisai, and Merck; has received payment or honoraria for OncLive and CurioScience; has received support for attending meetings and/or travel for Eisai and Tapimmune; has participated on Data Safety Monitoring boards or advisory boards for Sonnet Biotherapeutics and Seagen; and owns stock or stock options for Ovation Diagnostics. L.D. received payment from and served on an advisory board for Aadi Biosciences. R.S. has received royalties or licenses from UpToDate and Elsevier; has received consulting fees from Merck, Eisai, Daiichi Sankyo, GlaxoSmithKline, Seagen/Pfizer, and AbbVie; has received support for attending meetings or travel for GlaxoSmithKline and Merck; and has held an unpaid leadership or fiduciary role for the International Society of Gynecologic Cancer board. P. Braly has received grants or contracts for clinical trial support from Merck, Tasaro, Immunogen, and SpringWorks. T.R. has received support for attending meetings and/or travel as an employee of the University of Cincinnati. S.S. received payment or honoraria for serving on speakers bureaus for AstraZeneca, AbbVie/Immunogen, and GlaxoSmithKline/Tesaro. J.L. has received support for the present manuscript from SpringWorks. M.J. has received support for the present manuscript as a former employee of SpringWorks; had patents planned, issued, or pending as a former employee of SpringWorks; and owned stock or stock options from SpringWorks and Precision Biosciences. R.D. has received support for the present manuscript and owns stock or stock options from SpringWorks. S.A. has received support for the present manuscript and owns stock or stock options from SpringWorks. S.C. has received support for the present manuscript; received support for attending meetings and/or travel; has patents planned, issued, or pending; owns stock or stock options; and has received equipment and medical writing support from SpringWorks. C.A. owns stock or stock options from SpringWorks. P.A.K. has received grants or contracts from Novartis, AstraZeneca, Pfizer, Eli Lilly, Bayer, Merck, GlaxoSmithKline, Tesaro, and Merck KGaA and has received consulting fees for serving on advisory boards from AstraZeneca, Bayer, GlaxoSmithKline, Alkermes, Kadmon, Bristol Myers Squibb, IMV, Repare, Artios, Mersana, Novartis, Cardiff Oncology, Schrodinger, Inc., Mural Oncology, Scorpion, and Nimbus. K.P., K.M., M.B., P.C., and P.B. declare that they have no conflicts of interest.

## ETHICS STATEMENT

This trial was conducted in accordance with the ethical principles derived from the Declaration of Helsinki and all applicable laws, regulations, and scientific guidelines. All the patients provided written informed consent before enrolment.

## Data Availability

SpringWorks Therapeutics, Inc. is committed to data transparency and sharing data to further research while maintaining the privacy and confidentiality of research participants. Pertinent patient‐level data from completed registrational clinical trials will be made available by SpringWorks Therapeutics, Inc. to qualified researchers upon approval of reasonable requests following deidentification/anonymisation pursuant to applicable law. Requests for data must be sent to medinfo@springworkstx.com.
